# Supplementary material for: Direct laser-written optomechanical membranes in fiber Fabry-Perot cavities
Source: Nat Commun. 2024 Jan 3;15:209. doi: 10.1038/s41467-023-44490-7 (PMC10764917; doi:10.1038/s41467-023-44490-7)
Supplement: Supplementary file 1 — Supplementary Information [file 41467_2023_44490_MOESM1_ESM.pdf]

Supplementary figures to:
Direct laser-written optomechanical membranes in fiber Fabry-Perot cavities

Lukas Tenbrake [1,*] Alexander Faßbender,[2,*] Sebastian Hofferberth [1] Stefan Linden [2] and Hannes Pfeifer [1,†]

[1] *Institute of Applied Physics, University of Bonn, Germany*
[2] *Institute of Physics, University of Bonn, Germany*

This supplementary material comprises two additional figures relevant to the Methods section of the manuscript titled "Direct laser-written optomechanical membranes in fiber Fabry-Perot cavities".

Supplementary to Methods: Experimental setup

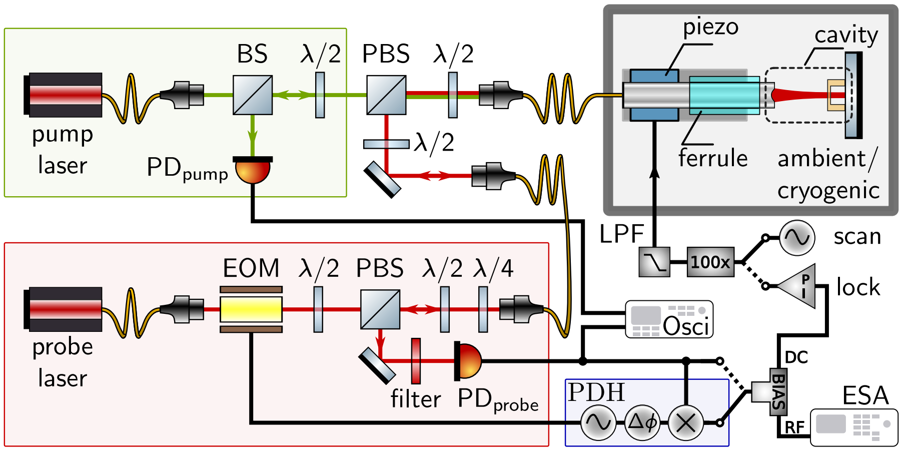

Supplementary Figure 1. Overview of the experiment setup. The green highlighted pump laser is only used within the optomechanical spring measurements. The optical cavity with the mechanical membrane resonator (gray box) is either located in ambient conditions (single fiber mirror and DBR substrate) or situated on the sample holder of a liquid Helium flow cryostat (two fiber mirrors in a glass ferrule). The optical cavity length can either be scanned or locked to the probe laser (highlighted in red) using either a Pound-Drever-Hall-type (highlighted in blue) or a side-of-fringe lock. For this purpose and as a frequency-meter in case of a scanned cavity, an electro-optic modulator is used to create sidebands to the main probe laser tone at adjustable RF-frequency up to 12 GHz. The photodiode reflection signals and the electrical noise spectrum of the lock can be measured using conventional oscilloscopes and an electric spectrum analyzer.

Supplementary to Methods: Optical properties of the membrane-cavity system

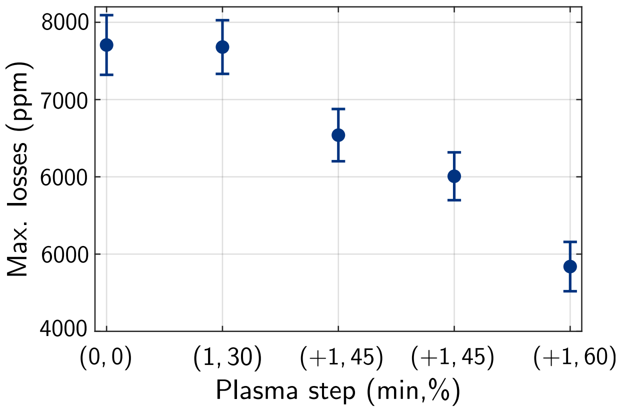

Supplementary Figure 2. Effect of oxygen plasma polishing on the amount of surface scattering from a DLW-fabricated thin film. The cavity losses are determined by measuring the minimal optical finesse of a polymer block structure with a height variation placed directly on a mirror substrate. Evaluating at the thickness of minimal finesse thereby ensures an intensity maximum of the intra-cavity field at the scattering polymer interface. The reduction of losses can therefore be attributed to the surface losses only, whilst their absolute value contains additional losses from the imperfections in the large bulk block below. In each step the same polymer structure underwent subsequent plasma steps of duration t_{polish} (minutes) at plasma power P_{plasma} (%) yielding the horizontal axis sets $(t_{\mathrm{polish}}, P_{\mathrm{plasma}})$. The error bars represent the combined fit and measurement uncertainty.

[*] These authors contributed equally to this work.
[†] hannes.pfeifer@iap.uni-bonn.de,
Current address: Department of Microtechnology and Nanoscience, Chalmers University of Technology, Gothenburg, Sweden
